# Perceived Knowledge of HIV-Negative Status Increases Condom Use Among Female Sex Workers in Zambian Transit Towns

**DOI:** 10.1089/apc.2019.0266

**Published:** 2020-04-21

**Authors:** Katrina F. Ortblad, Michael M. Chanda, Magdalene Mwale, Jessica E. Haberer, Margaret McConnell, Catherine E. Oldenburg, Till Bärnighausen

**Affiliations:** ^1^ Department of Global Health, University of Washington, Seattle, Washington, USA.; ^2^ John Snow, Inc., Lusaka, Zambia.; ^3^ Department of General Internal Medicine, Massachusetts General Hospital Global Health, Boston, Massachusetts, USA.; ^4^ Department of Global Health and Population, Harvard T.H. Chan School of Public Health, Boston, Massachusetts, USA.; ^5^ Francis I. Proctor Foundation, University of California, San Francisco, San Francisco, California, USA.; ^6^ Department of Ophthalmology, University of California, San Francisco, San Francisco, California, USA.; ^7^ Department of Epidemiology and Biostatistics, University of California, San Francisco, San Francisco, California, USA.; ^8^ Africa Health Research Institute (AHRI), Somkhele and Durban, South Africa.; ^9^ Heidelberg Institute of Public Health (HIGH), University of Heidelberg, Heidelberg, Germany.

**Keywords:** HIV, testing, key and vulnerable populations, sex workers, women, sexual behaviors

## Abstract

Knowledge of HIV status is a necessary pre-condition for most HIV interventions, including treatment as well as biomedical and behavioral prevention interventions. We used data from a female sex worker (FSW) cohort in three Zambian transit towns to understand the effect that knowledge of HIV status has on FSWs' HIV risk-related sexual behaviors with clients. The cohort was formed from an HIV self-testing trial that followed participants for 4 months. Participants completed three rounds of data collection at baseline, 1 month, and 4 months where they reported their perceived knowledge of HIV status, number of clients on an average working night, and consistent condom use with clients. We measured the effect of knowledge of HIV status on participants' sexual behaviors by using linear regression models with individual fixed effects. The majority of the 965 participants tested for HIV at least once during the observation period (96%) and changed their knowledge of HIV status (79%). Knowledge of HIV status did not affect participants' number of clients, but it did affect their consistency of condom use. Compared with unknown HIV status, knowledge of HIV-negative status significantly increased participants' consistent condom use by 8.1% points [95% confidence interval (CI): 2.7–13.4, *p* = 0.003] and knowledge of HIV-positive status increased participants' consistent condom use by 6.1% points (95% CI: −0.1 to 12.9, *p* = 0.08); however, this latter effect was not statistically significant. FSWs in Zambia engaged in safer sex with clients when they learned their HIV status. The expansion of HIV testing programs may serve as a behavioral HIV prevention measure among FSWs.

## Introduction

Despite numerous studies in diverse populations, the effect of knowledge of HIV status on sexual behaviors associated with an increased risk of HIV transmission remains unclear.^1–11^ The efficacy and effectiveness of a range of biomedical and behavioral HIV interventions on HIV transmission, however, are firmly established.^12–23^ Knowledge of HIV status is a necessary pre-condition for most HIV interventions and thus HIV testing is a priority for HIV prevention interventions such as treatment as prevention (TasP)^14,15^ and pre-exposure prophylaxis (PrEP).^18,19,24–27^

Many governments and international organizations are currently investing heavily in the expansion of HIV testing services in high HIV prevalence settings,^28,29^ including clinic-based testing,^28^ home-based testing,^30,31^ and, recently, HIV self-testing.^32–34^ Although regular HIV testing is important for all individuals in high HIV prevalence settings, it is especially important for individuals engaging in behaviors that put them at particular risk of HIV infection, such as female sex workers (FSWs) and men who have sex with men.

Previous studies among members of the general population in high HIV prevalence settings largely suggest that knowledge of HIV-negative status does not affect HIV risk-related sexual behaviors,^2,3,6–10^ whereas knowledge of HIV-positive status decreases HIV risk-related sexual behaviors.^1,2,4,5,7,8,10^ The assumption here is that individuals who learn they are not living with HIV do not change their sexual behavior because they do not perceive themselves at risk of infection, whereas those who learn they are living with HIV change their behavior to protect their sexual partners from becoming HIV infected.

This assumption, however, might not hold true for FSWs who have strong economic incentives for multiple sexual partners and the provision of condomless sex; they may additionally assume that many of their partners are already living with HIV. A recent study among an FSW cohort in Kampala, Uganda, for example, found that FSWs in this setting increased condom use with clients when they learned they were not living with HIV (presumably to prevent infection) and decreased condom use with clients when they learned they were living with HIV (presumably to earn more income).^35^

With this study, we aim to understand the effect of knowledge of HIV status on HIV risk-related sexual behavior in a different cohort of FSWs that consists of individuals from three different Zambian transit towns: Livingstone, Chirundu, and Kapri Moposi. The FSWs in this setting differ from those in Kampala, because their clientele are primarily transient truck drivers and, at the time of the study, no health services catered to FSWs were available at these locations. Understanding the effect of knowledge of HIV status on FSWs' sexual behaviors with clients is important for catering the counseling messages delivered with HIV testing services, especially if FSWs might engage in greater sexual risk taking with knowledge of HIV-positive status.

## Materials and Methods

### Study settings

Livingstone, Chirundu, and Kapiri Mposhi are three Zambian transit towns where trucks traveling throughout southern Africa are required to stop. Livingstone and Chirundu are on the Zambia-Zimbabwe border, and in Kapiri Mposhi, north of this border, there is a weigh station.^36^ The sex industry in these towns is primarily driven by the trucking industry and the tourism industry in Livingstone, the site of Victoria Falls.^36,37^ In Zambia, one in two FSWs is estimated to be living with HIV.^28^

### Participants

From September to November 2016, the FSWs in this study cohort were enrolled in a three-armed cluster-randomized trial of different peer-based HIV self-testing delivery models (ClinicalTrials.gov registration no. NCT03517566).^33^ Participants were recruited by FSW peer educators and assessed for eligibility by research assistants. Eligible participants were: (1) 18 years of age or older, (2) exchanged sex for money or goods at least once in the past month, and (3) reported never testing for HIV or testing HIV negative at their last test (more than 3 months earlier).

Over the 4-month duration of the trial, participants in the cohort completed four peer educator visits (at month 0, 0.5, 1.5, and 3). At each of these visits, peer educators provided participants with information on HIV prevention, condoms, and the locations for nearby HIV testing (using standard services). For participants in the HIV self-testing intervention arms, peer educators additionally delivered either an HIV self-test or a coupon, exchangeable for an HIV self-test at a nearby health care clinic, at the first and fourth peer educator visit (months 0 and 3). At study completion (month 4), all participants were given the opportunity to test for HIV by using a blood-based rapid test.

Institutional review boards at the Harvard T.H. Chan School of Public Health (USA) and ERES Coverage (Zambia) granted ethical approval for the trial. We obtained written informed consent from all participants.

### Rounds of data collection

Participants completed three rounds of data collection at baseline, 1 month, and 4 months. Research assistants collected electronic data (CommCare, Dimagi, Inc., Cambridge, MA) using face-to-face interviews at private locations selected by participants (e.g., empty bar, home, guest house).

### Outcome: sexual behaviors

We measured the effect of knowledge of HIV status on two sexual behavior outcomes: (1) participants' number of clients on an average working night, and (2) participants' consistent condom use with clients. At all rounds of data collection, participants were asked to report the number of clients they have sex with on an average working night and the number of these with whom they use a condom. If participants reported using condoms with all their clients, their condom use was categorized as consistent.

### Exposure: knowledge of HIV status

In this study, we categorized participants' knowledge of HIV status into three different knowledge states: (1) knowledge of HIV-negative status, (2) knowledge of HIV status unknown, and (3) knowledge of HIV-positive status. At each round of data collection, participants were asked to report the likelihood that they were currently living with HIV by using a 10-rung ladder scale (higher rungs equated to higher likelihood of HIV infection). Participants' responses largely lumped around 1, 5, and 10; thus, we categorized knowledge of HIV status as negative (rungs 1–3), unknown (rungs 4–7), and positive (rungs 8–10). This measurement approach has previously been used in other studies, including a similar study among FSWs in Uganda.^35^

Our measurement of knowledge of HIV status in this study is participants' perceived knowledge of HIV status, which may not reflect participants' most recent HIV test result. Among our study participants, perceived HIV status may differ from actual HIV status for reasons including a new HIV risk encounter after recent HIV testing, mistrust of a new HIV testing technology (e.g., self-testing), and participation in an intervention believed to cure HIV infection (e.g., religious ceremony).^38–40^ For these reasons, we believe that perceived knowledge of HIV status is more likely to affect sexual behavior than actual HIV status; we therefore included it as the exposure variable in our analysis. In addition, perceived knowledge of HIV status versus actual HIV status can be updated with an intervention—regular HIV testing—over time.

### Covariates

We measured participants' sociodemographic characteristics at baseline and recent HIV testing—including self-reported HIV test results—at each study round (reflecting the past 3 months at baseline, and past month at 1 and 4 months). Our electronic data collection platform automatically captured the round of data collection (e.g., baseline, 1 month, 4 months) and calendar month of observation (e.g., January, March). We recorded the results of the rapid HIV test, conducted among willing participants, at 4 months.

### Effect size estimation

We used longitudinal data and fixed-effects estimation to measure the effect of knowledge of HIV status on FSWs' sexual behaviors with clients. We used individual fixed effects to control for all—both observed and unobserved—individual-level confounding that did not vary over the study observation period (including genetic makeup, stable psychological traits, and ethnic, religious, and social backgrounds). We used survey round fixed effects to additionally control for changes shared by the FSWs that were induced by the surveys and accompanying interactions with study staff. Finally, we used calendar time fixed effects to control for confounding factors that were shared by all FSWs in our study (including background policy changes, background health systems reforms, and public campaigns).^41,42^

As a result of these fixed effects, we thus controlled for major categories of unobserved confounding factors—time-constant individual characteristics and round- and time-varying factors shared by all FSWs— implying that our results may be relatively strongly causal.^42^ We adjusted our standard errors (SEs) for clustering at the level of the peer educator, because peer educators were involved in the recruitment of participants and trained participants on how to interpret HIV self-test results.

### Sensitivity analyses

We conducted three sensitivity analyses to confirm the effect of knowledge of HIV status on FSWs' sexual behaviors with clients. First, we tested the robustness of our linear regression models with Poisson regression models for the number of clients per average working night and logistic regression models for consistent condom use with clients. These models included individual-level fixed effects, control variables for round of data collection and calendar month, and clustered SEs at the level of the peer educator.

Second, we tested the effect of controlling for potential time-varying confounders (i.e., round of data collection and calendar month) by running unadjusted linear regression models with individual-level fixed effects and SEs clustered at the level of the peer educator.

Third, to understand the effect of knowledge of HIV status on sexual behavior among those who likely changed their knowledge as a result of HIV testing, we limited our sample to participants who self-reported testing for HIV since the first round of data collection (month 0) and estimated effect sizes by using the adjusted linear regression models from the main analyses.

### Sub-group analyses

To understand how different changes in knowledge of HIV status (i.e., changing knowledge of HIV status from unknown to HIV-negative or from HIV-negative to HIV-positive) affect FSWs' HIV risk-related sexual behaviors, we conducted a number of sub-group analyses. We divided participants into six sub-groups based on their baseline knowledge of HIV status (e.g., HIV-negative; unknown; HIV-positive) and HIV risk-related sexual behaviors (e.g., average number of clients below vs. equal and above median number; consistent vs. inconsistent condom use).

By dividing participants by their knowledge of HIV status at baseline, we can better understand how specific changes in knowledge from prior knowledge states affect sexual behavior. Then, by dividing participants by their HIV risk-related sexual behaviors at baseline, we can better understand changes in sexual behavior following knowledge of HIV status among those who had room to change their sexual behavior. For example, those who were already engaging in consistent condom use at baseline are unlikely to change condom use later with knowledge of HIV-negative status. We used the linear regression models with individual-level fixed effects described earlier for all sub-group analyses.

Stata 13.1 (StataCorp, College Station, TX) was used to conduct all analyses in this article.

## Results

From September to November 2016, 1280 potential participants were screened and 965 were eligible and enrolled in the FSW cohort: 480 (50%) in Livingston, 240 (25%) in Chirundu, and 245 (25%) in Kapiri Mposhi.^33^ The median age of participants was 25 years (interquartile range: 21–30) (Table 1). At baseline, participants reported a mean number of 4.7 clients (standard deviation: 7.4) on an average working night and 26% (245/941) of participants reported consistent condom use with clients. Loss to follow-up over the 4-month study duration was low: 8% (79/965) at 1 month and 7% (67/965) at 4 months. By 4 months, almost all participants (95%, 859/898) had tested for HIV at least once since the start of the study.

**Table 1.** Participant Characteristics^a^

Age (median, IQR)	25 (21–30)
Education	
No formal	108/964 (11.2%)
Primary/junior	450/964 (46.7%)
Secondary	389/964 (40.4%)
Vocational	13/964 (1.4%)
Tertiary	4/964 (0.4%)
Monthly income, USD^b^	
No income	202/949 (21.3%)
<$30	123/949 (13.0%)
$30–$60	235/949 (24.8%)
$60–$125	246/949 (25.9%)
>$125	143/949 (15.1%)
Timing of last HIV test	
>3–6 months	377/948 (39.8%)
>6–12 months	240/948 (25.3%)
>12–24 months	70/948 (7.4%)
>24 months	65/948 (6.9%)
Never tested	196/948 (20.7%)
Of 10 clients, no. who think they are HIV-positive (median, IQR)	8 (5–9)
Price for vaginal sex, USD^b^ (mean, SD)	
With a condom	$10.14 ($7.03)
Without a condom	$19.37 ($13.74)
No. of clients/average night (median, SD)	4.7 (7.4)
Consistent condom use with clients^c^	245/941 (26.0%)
Tested for HIV, since the start of the study^d^	
1 month	790/885 (89.3%)
4 months	859/898 (95.7%)

^a^ All characteristics and behaviors measured at baseline with the exception of testing for HIV since the start of the study.

^b^ Price categories in USD; October 10th, 2016 exchange rate (1 USD = 9.93 Zambian Kwatcha). Categories are the estimates for the average conversion rate in both countries.

^c^ The use of condoms with all clients that female sex workers have sex with on an average working night.

^d^ Loss to follow-up was 8% (79/965) at 1 month and 7% (67/965) at 4 months.

IQR, interquartile range; SD, standard deviation; USD, US dollars.

**FIG. 1.**
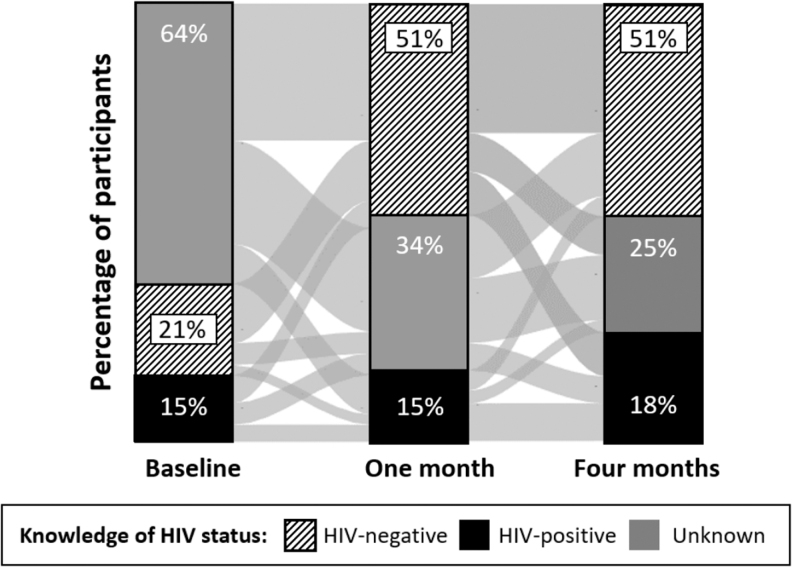
Participants' knowledge of HIV status at baseline, 1 month, and 4 months. Knowledge of HIV-negative status (stripes); knowledge of HIV-positive status (black); unknown HIV status (gray). Lines between the bars show how participants changed their knowledge of HIV status over the duration of the study.

The majority (79%, 645/813) of participants who completed three rounds of data collection changed their knowledge of HIV status over the 4-month duration of the study—indicated by the gray horizontal lines going between the vertical bars in Fig. 1. Among those who changed their knowledge of HIV status, 98% (634/645) tested for HIV at least once since the start of the study. At baseline, knowledge of HIV status was unknown to the majority of participants (64%, 610/956), but then by 1 month and 4 months knowledge of HIV status was HIV-negative for roughly half of participants (1 month: 51%, 450/879; 4 months: 51%, 453/882). By 4 months, knowledge of HIV status was HIV-positive for roughly a quarter of participants (24%, 208/882).

**FIG. 2.**
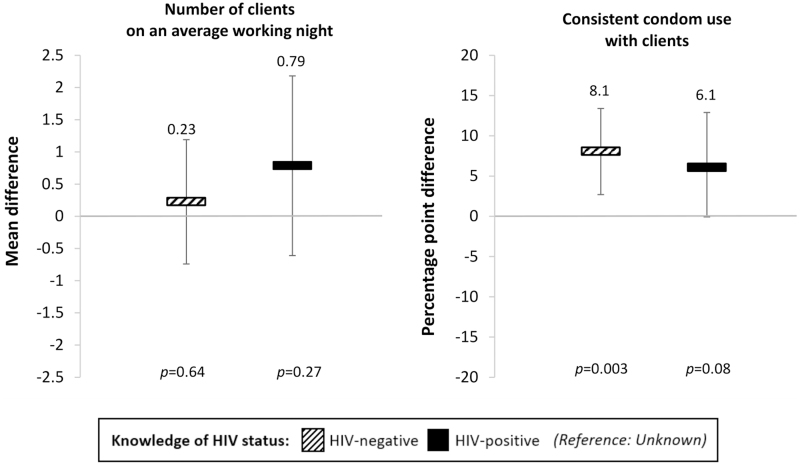
The effect of knowledge of HIV status on female sex workers' sexual behaviors with clients. We measured effect size estimates by using linear panel regressions with fixed effects for individuals, round of data collection, and calendar month, and standard error clustered at the level of the peer educator. Striped bars: knowledge of HIV-negative status; black bars: knowledge of HIV-positive status (reference category: knowledge of HIV status unknown). Black vertical lines indicate 95% confidence intervals.

Knowledge of HIV status did not affect participants' number of clients on an average working night, but it did affect consistent condom use with clients (Fig. 2). Compared with knowledge of HIV status unknown, knowledge of HIV-negative status significantly increased participants' consistent condom use with clients by 8.1% points [95% confidence interval (CI): 2.7–13.4, *p* = 0.003], whereas knowledge of HIV-positive status non-significantly increased participants' consistent condom use by 6.1% points (95% CI: −0.1 to 12.9, *p* = 0.08). These findings remained largely consistent in the three sensitivity analyses (Appendix Table 1); the only main difference was that knowledge of HIV-positive status significantly increased participants' number of clients on an average working night in the first sensitivity analysis, which used Poisson regression models (incidence rate ratio: 1.15, 95% CI: 1.08–1.23, *p* < 0.001).

**FIG. 3.**
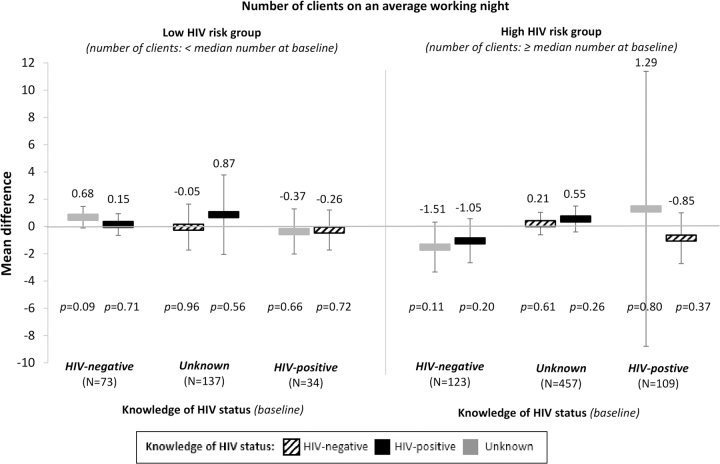
The effect of changes in knowledge of HIV status on female sex workers' number of clients, from baseline knowledge and sexual behavior. We divided participants into sub-groups by their knowledge of HIV status and HIV risk-related sexual behaviors at baseline (i.e., low risk and high risk). The reference for each sub-group is participants' knowledge of HIV status at baseline. We measured effect size estimates by using linear panel regressions with fixed effects for individuals, round of data collection, and calendar month, and standard error clustered at the level of the peer educator. The bars show the mean differences in the number of clients for participants whose knowledge of HIV status changed from different states at baseline (listed by sub-group along the x-axis) to HIV-negative (striped bars), HIV-positive (black bars), or unknown (gray bars). Black vertical lines indicate 95% confidence intervals.

**FIG. 4.**
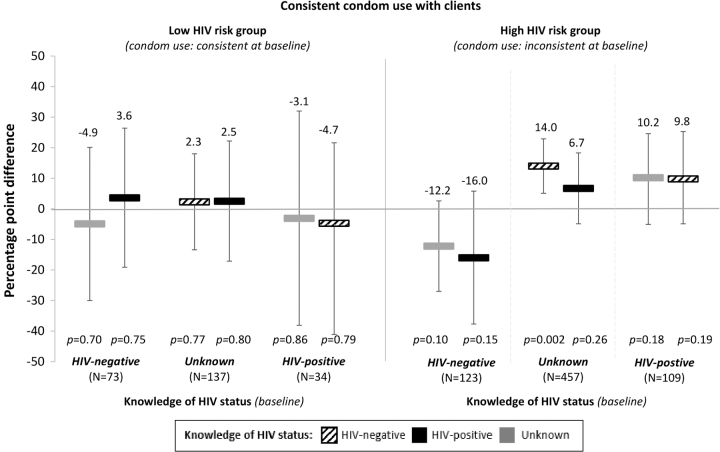
The effect of changes in knowledge of HIV status on female sex workers' condom use with clients, from baseline knowledge and sexual behavior. We divided participants into sub-groups by their knowledge of HIV status and HIV risk-related sexual behaviors at baseline (i.e., low risk and high risk). The reference for each sub-group is participants' knowledge of HIV status at baseline. We measured effect size estimates by using linear panel regressions with fixed effects for individuals, round of data collection, and calendar month, and standard error clustered at the level of the peer educator. The bars show the average percentage-point differences in the probability of consistent condom use for participants whose knowledge of HIV status changed from different states at baseline (listed by sub-group along the x-axis) to HIV-negative (striped bars), HIV-positive (black bars), or unknown (gray bars). Black vertical lines indicate 95% confidence intervals.

In the sub-group analyses that measured how different changes in knowledge of HIV status affected participants' HIV risk-related sexual behaviors, we found no effect of knowledge of HIV status on participants' number of clients on an average working night (Fig. 3) and some larger effects of knowledge of HIV status on participants' consistent condom use with clients (Fig. 4). Among participants who did not consistently use condoms and whose knowledge of HIV status was unknown at baseline, knowledge of HIV-negative status significantly increased consistent condom use by 14.0% points (95% CI: 5.1–22.9, *p* = 0.002) and knowledge of HIV-positive status non-significantly increased consistent condom use by 6.7% points (95% CI: −4.9 to 18.3, *p* = 0.26).

## Discussion

Among FSWs in three Zambian transit towns, knowledge of HIV status did not affect FSWs' number of clients per average working night, but it did increase FSWs' consistent condom use with clients. Both knowledge of HIV-negative and HIV-positive status increased FSWs' consistent condom use compared with knowledge of HIV status unknown; however, the increases in consistent condom use with knowledge of HIV-positive status were not significant (*p* = 0.08).

The finding that knowledge of HIV-negative status significantly increases consistent condom use with clients is consistent with the findings from a similar study among a cohort of FSWs in urban Uganda,^35^ but it contradicts much of the literature among members of the general population suggesting that knowledge of HIV-negative status does not affect HIV risk-related sexual behaviors.^2,3,6–10^ The suggestion that knowledge of HIV-positive status might also increase consistent condom use with clients is contradictory to the findings from the similar study among the Ugandan FSW cohort (which suggest that knowledge of HIV-positive status may decrease consistent condom use with clients),^35^ but it is consistent with literature among members of the general population.^1,2,4,5,7,8,10^

The observed increase in consistent condom use following knowledge of HIV-negative status is encouraging, as it indicates that FSWs in this setting are incentivized to engage in behaviors that decrease their risk of HIV acquisition. In doing so, FSWs in this population are foregoing the opportunity to make double the amount for sex without a condom compared with sex with a condom. This ability to forgo condomless sex with clients suggests that FSWs in this setting have the power to negotiate condom use and thus is contrary to literature, suggesting that FSWs are powerless in their sexual relationships.^43,44^ In addition, FSWs in this setting do not appear to be making up for lost income by increasing their number of clients per average working night. These findings thus support interventions targeted at increasing FSW agency, so that they are empowered to engage with clients in a way that best serves both their economic and health needs.

The observed, but non-significant, increase in consistent condom using following knowledge of HIV-positive status is also encouraging, as it indicates that FSWs in this setting are incentivized to engage in behaviors that decrease their clients' risk of HIV acquisition. It is somewhat surprising that FSWs in this setting that perceived themselves to be living with HIV increased consistent condom use with clients, considering that they also perceived the vast majority of their clients to be living with HIV. It is possible that few FSWs are having conversations about HIV status disclosure with their clients, and thus in a state of uncertainty, err on the side of caution for their clients' health. Still, it is important to emphasize the necessity of continued condom use among FSWs after HIV diagnosis to prevent HIV transmission to clients and infection of sexually transmitted infections or different HIV strains to FSWs.

The overall protective effect of knowledge of HIV status on HIV risk-related sexual behaviors among FSWs supports the expansion of HIV testing interventions among members of this population. At the time of this study, a few FSW-specific health services were available to FSWs working in this setting, which is contrary to other countries, such as Uganda, that have large initiatives dedicated to providing free health services to FSW and other high HIV risk populations (i.e., through the Most at Risk Populations Initiative).

In this study, the strongest protective effects of knowledge of HIV status on FSWs' HIV risk-related sexual behaviors were among FSWs whose knowledge of HIV status was unknown and whose risk of HIV acquisition was high at baseline. Thus, novel HIV testing interventions that target these individuals—such as venue-based HIV testing, specific FSW-friendly health care clinics, or peer-distributed HIV self-testing (like that in this study)—should be considered to increase HIV testing among and improve the overall health of FSWs in this setting. Since consistent condom use is difficult, even with updated knowledge of HIV status, HIV testing interventions targeted at FSW should be linked with other prevention interventions—such as PrEP—that offer protection even in the absence of condom use.^45–47^

This study had a number of strengths. First, the sample size was large and drawn from three different Zambian transit towns, increasing the generalizability of study results in Zambia and potentially other similar settings. Second, there was little loss to follow-up and the majority of FSWs changed their knowledge of HIV status over the study period (almost all of which did so through recent HIV testing), which allowed for a large sample to which the individual fixed-effects estimation approach could be applied. Third, the individual fixed-effects estimation approach is a rigorous quasi-experimental method that allows for stronger causal inference than analysis of cross-sectional data,^41,42^ thus increasing the strength of our findings beyond that of previous studies.

This study also had some limitations. First, all sexual behavior outcomes were self-reported by participants and thus subject to social desirability bias.^48^ Participation in the trial included access to free condoms and peer education counseling on HIV prevention, thus participants may have falsely reported consistent condom use with clients at follow-up because they were receiving interventions that clearly encouraged this behavior. This bias would have inflated the positive observed effects that knowledge of HIV-negative and HIV-positive status had on FSWs' consistent condom use with clients. Second, we followed participants for a duration of 4 months only. However, we do not expect this period of follow-up to affect our findings because if changes in sexual behaviors were to occur with new knowledge of HIV status, we would expect this to happen shortly after the change in knowledge of HIV status took place. Third, we only measured the effect of knowledge of HIV status on FSWs' sexual behaviors with clients. Knowledge of HIV status may differentially affect FSWs' sexual behaviors with non-clients, especially if FSWs are more confident in these sexual partners' HIV status. Finally, FSW populations across sub-Saharan Africa are diverse,^49,50^ and thus the generalizability of these findings to other sub-Saharan FSW populations may be limited.

## Conclusions

The World Health Organization and many sub-Saharan African countries recommend frequent testing among FSWs to detect early HIV infection and prevent HIV transmission.^51^ Our results indicate that frequent HIV testing and subsequent changes in knowledge of HIV status will also affect FSWs' HIV risk-related sexual behaviors in ways that further prevent HIV infection. Specifically, knowledge of HIV negative status significantly increased FSWs' consistent condom use with clients. Despite high HIV risk, the majority of FSWs that test for HIV will test HIV-negative. Thus, our findings support the expansion of HIV testing programs and linked HIV prevention interventions (e.g., PrEP) for FSWs in this and similar settings.

## Appendix

**Appendix Table 1.** Sensitivity Analyses: The Effect of Knowledge of HIV Status on HIV Risk-Related Sexual Behaviors

		HIV risk-related sexual behaviors			
	Knowledge of HIV status^a^	Number of clients/average night		Consistent condom use^b^	
		**IRR^3^ (95% CI)**	***p***	**OR^3^ (95% CI)**	***p***
Sensitivity analysis 1: Poisson and logistic regression models^c^	Unknown	ref		ref	
	HIV-negative	1.04 (0.98 to 1.10)	0.23	1.50 (1.10 to 2.04)	0.01
	HIV-positive	1.15 (1.08 to 1.23)	<0.001	1.32 (0.92 to 1.95)	0.13
		**MD^4^ (95% CI)**	***p***	**PP^4^ (95% CI)**	***p***
Sensitivity analysis 2: Unadjusted linear regression models^d^	Unknown	ref		ref	
	HIV-negative	−0.18 (−0.87 to 0.52)	0.62	0.18 (0.12 to 0.23)	<0.001
	HIV-positive	0.47 (−0.76 to 1.69)	0.46	0.12 (0.06 to 0.19)	<0.001
		**MD^5^ (95% CI)**	***p***	**PP^5^ (95% CI)**	***p***
Sensitivity analysis 3: Among those who reported testing since start of study^e^	Unknown	ref		ref	
	HIV-negative	0.08 (−0.87 to 1.04)	0.87	7.4 (1.7 to 13.2)	0.01
	HIV-positive	0.72 (−0.60 to 2.04)	0.28	4.6 (−2.3 to 11.4)	0.19

^a^ Participants were asked to report the likelihood they currently had HIV on a 1–10 scale: HIV-negative status (1–3), unknown HIV status (4–7), and HIV-positive status (8–10).

^b^ Categorized as not using condoms with at least one client on an average working night.

^c^ Effect size estimates measured using longitudinal data and Poisson (number of clients) or logistic (consistent condom use) regression models with individual effects for individuals, round of data collection, and month; standard errors clustered at the level of the peer educator.

^d^ Effect size estimates measured using longitudinal data and regression models with individual effects for individuals; standard errors clustered at the level of the peer educator.

^e^ Effect size estimates measured using longitudinal data and regression models with individual effects for individuals, round of data collection, and month; standard errors clustered at the level of the peer educator.

CI, confidence interval; FSW, female sex worker; IRR, incidence rate ratio; MD, mean difference; OR, odds ratio; *p*, *p* value; PP, percentage point change.
